# Preparation and Characterization of New Geopolymer-Epoxy Resin Hybrid Mortars

**DOI:** 10.3390/ma6072989

**Published:** 2013-07-17

**Authors:** Francesco Colangelo, Giuseppina Roviello, Laura Ricciotti, Claudio Ferone, Raffaele Cioffi

**Affiliations:** Department of Engineering, University of Naples ‘Parthenope’, INSTM Research Group Naples Parthenope, Centro Direzionale Naples, Isola C4, 80143 Naples, Italy; E-Mails: giuseppina.roviello@uniparthenope.it (G.R.); laura.ricciotti@uniparthenope.it (L.R.); claudio.ferone@uniparthenope.it (C.F.); raffaele.cioffi@uniparthenope.it (R.C.)

**Keywords:** geopolymer mortar, epoxy resin, hybrid composites, metakaolin, interfacial transition zone

## Abstract

The preparation and characterization of metakaolin-based geopolymer mortars containing an organic epoxy resin are presented here for the first time. The specimens have been prepared by means of an innovative *in situ* co-reticulation process, in mild conditions, of commercial epoxy based organic resins and geopolymeric slurry. In this way, geopolymer based hybrid mortars characterized by a different content of normalized sand (up to 66% in weight) and by a homogeneous dispersion of the organic resin have been obtained. Once hardened, these new materials show improved compressive strength and toughness in respect to both the neat geopolymer and the hybrid pastes since the organic polymer provides a more cohesive microstructure, with a reduced amount of microcracks. The microstructural characterization allows to point out the presence of an Interfacial Transition Zone similar to that observed in cement based mortars and concretes. A correlation between microstructural features and mechanical properties has been studied too.

## 1. Introduction

The term “geopolymer” was first used by J. Davidovits in the late 1970s and nowadays identifies a family of amorphous alkali or alkali-silicate activated aluminosilicate binders of composition M_2_O·*m*Al_2_O_3_·*n*SiO_2_, usually with *m* ≈ 1 and 2 ≤ *n* ≤ 6 (M usually is Na or K) [1].

The synthesis of geopolymers takes place starting from reactive precursors such as metakaolin (kaolinite calcined at 600–700 °C) or many other natural and artificial silico-aluminates, which are mixed with alkali metal (Na or K) hydroxide and/or silicate solutions [2,3,4,5,6,7,8]. When in contact with a high pH alkaline solution, aluminosilicate reactive materials release free SiO_4_ and AlO_4_ tetrahedral units which afterwards condensate to form a rigid network.

Geopolymer based materials are attractive because excellent mechanical properties, high early strength, freeze-thaw resistance, low chloride diffusion rate, abrasion resistance, thermal stability and fire resistance, can be achieved [9,10,11,12]. Due to their lower calcium content, they are more resistant to acid attack than Portland cement based materials [13]. In addition, geopolymer based materials are of great interest because of the reduced energy requirement for their manufacture. In fact, the reaction pathway requires either metakaolin or raw silico-aluminates so that greenhouse gas emissions can be reduced up to 80% in comparison to traditional cement based materials [11,14]. In fact, even if natural aggregates are substituted by sustainable artificial ones, manufactured by using industrial wastes [15,16,17,18,19], the emissions of CO_2_ in concrete industry is mainly linked to the use of ordinary Portland cement as a binder.

Applications of geopolymer-based materials in the fields of new ceramics, binders, matrices for hazardous waste stabilization, fire-resistant materials, asbestos-free materials, and high-tech materials have been documented [20,21,22,23,24].

In order to improve several properties of geopolymer materials, such as their brittle behavior and their low flexural strength, which usually limit their extensive applications as a structural material, geopolymer composites have been introduced in the last years. Geopolymer based composites are usually obtained by incorporation of organic polymers [25,26,27,28,29,30], such as polyvinyl acetate, polypropylene, polyvinyl alcohol, or water-soluble organic polymers [31,32,33,34,35].

Recently [36], the authors have prepared novel organic-inorganic hybrid composites, adding epoxy resins to geopolymer, through an innovative synthetic approach based on a co-reticulation in mild conditions of organic and inorganic components, producing an intimate and homogeneous dispersion of the organic phase into the inorganic one. Through this novel synthetic approach, polymeric composites are not obtained by simply adding the organic polymer as fibers or emulsion as usually reported in the literature, but the resin is allowed to crosslink *in situ* during the geopolymerization reaction. In this way, the use of compatibilizers is fully avoided [37,38,39,40]. These new materials, highly homogeneous up to micrometric level even at appreciable concentration of resin (up to 20% w/w), show good technological properties: in particular, in respect to the neat geopolymer, they present significantly enhanced compressive strength and toughness, suggesting their possible practical applications in the manufacture of high durability composites (e.g., thermo-resistant), in the field of restoration and repairing of damaged concrete and masonry.

Another common problem of geopolymer based pastes representing an important barrier to their structural application is the high shrinkage that is observed during the curing process and the cracking of specimens caused by water loss under normal environmental conditions [41,42]. These phenomena may be strongly reduced by adding filler to the geopolymer. The addition of sand also contributes to the reduction of the cost of the final product.

Geopolymeric mortars are usually prepared by mechanically mixing alumino silicate materials, *i.e*., metakaolin (MK) or fly ash, with standard sand and an alkaline activating solution in appropriate proportions. However, addition of sand significantly alters the pore structure of the material, which in turn strongly influences the properties of the geopolymeric mortar [43]. In addition, the workability of the fresh mixture is greatly influenced by the addition of sand [44].

In order to face the issues previously described and, at the same time, to improve the technological properties of the geopolymeric mortars, in this paper novel geopolymeric mortars containing epoxy resins with different sand content have been produced. Their physical and mechanical performances have been compared with neat geopolymer MK-based mortar and pastes. Moreover, a detailed microstructural analysis has been performed in order to investigate the morphology and the interfacial interactions between the geopolymeric-organic matrix and the fine aggregates.

Finally, it is worth pointing out that, to the best of our knowledge, this paper represents the first attempt to produce and characterize hybrid geopolymer-epoxy resin mortars.

## 2. Results and Discussion

### 2.1. Preparation Method

Novel hybrid geopolymeric mortars containing Epojet^®^ and EpojetLV^®^ resins were prepared, and their physical-mechanical properties were compared with the geopolymeric mortar specimen and with the corresponding pastes. Epojet^®^ is a two-component epoxy adhesive for injection, which, after mixing, becomes a low viscosity liquid. According to the producer technical data sheet [45], it is usable for 40 min at room temperature. EpojetLV^®^ is a highly low-viscosity two-component epoxy adhesive for injection in microcracks which, after mixing, becomes a fluid liquid. With respect to Epojet^®^, EpojetLV^®^ takes a longer time to harden, since it is usable for approximately 70 min.

Three sets of mortars with different sand content were prepared and have been indicated with a letter (G for Geopolymer, E for the composite geopolymeric mortar/Epojet, L for the composite geopolymeric mortar/EpojetLV), and a number indicating the percentage of sand employed (no number to indicate 0% sand content, that is the paste [46]; 33 indicating 33.3% by weight of sand in respect to the total mass; 66 indicating 66.6% by weight of sand in respect to the total mass).

The hybrid mortars were prepared by adding the epoxy resin into the geopolymeric mortar under mechanical stirring, when both geopolymerization and organic polycondensation were initiated but not completed yet. This innovative approach, only very recently introduced [36], enables the production of hybrid materials characterized by a homogenous microdispersion of the resin particles into the inorganic matrix.

The incorporation of the resins into the geopolymeric mixture when both were partly reacted but still easily workable represents the key step of the procedure: in fact the addition of the unreacted components of the resins to the inorganic suspension produce an undesirable phase segregation phenomenon. On the contrary, a late mixing of the two components (the cured organic resin and the geopolymer mortar) strongly reduces the compatibility and the homogeneity of the dispersion of the two phases. For this reason, a careful execution of the synthetic procedure is essential in order to produce new materials with desirable properties.

In this experimental program, different amounts of epoxy resins into the geopolymeric mortar have been considered. Only the results obtained with a 20% of organic resin in respect to the paste weight are here reported, since a lower amount does not significantly affect the mechanical properties of the final material, while a larger amount produces relevant segregation phenomena [36,38].

### 2.2. Characterization

In the following sub-sections, the microstructural, thermal and physico-mechanical characterizations are reported and discussed. As far as X ray Diffraction (XRD) and Fourier Transformed Infra Red Spectroscopy (FT-IR) analyses, usually performed on inorganic polymer materials [47], are not here reported since the presence of crystalline silica masks the characteristic bands and signals of the amorphous material. However, in our previous work [36], a deep characterization of an analogous system without sand has been reported.

#### 2.2.1. Microstructural Analysis

The effect of the addition of the organic resin on the morphology of the geopolymeric mortar and on the interfacial zone between the matrix and the aggregate was investigated by means of optical and electronic microscopy. The microstructural analysis here discussed, refers to the specimens containing 66.6% by weight of sand. Different sand contents do not significantly alter the morphology of the samples.

**Figure 1:**
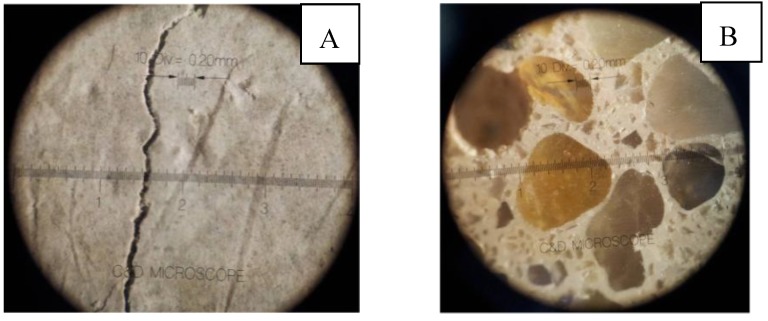
Crack Measurement Microscope images for (**A**) G66 (external surface); and (**B**) E66 (polished surface).

Systems containing organic resins do not show evident cracks, which are on the contrary present in the geopolymeric mortar prepared without resins. In Figure 1, optical microscope images of systems G66 (A) and E66 (B) are reported. The former is taken on the plane specimen to better observe the actual fractured external surface, while the latter is taken on a polished surface to check the textural homogeneity of the mortar. As shown in the figure, the organic phase plays an important role in producing compact geopolymer mortar. In fact, the visible microcracks of the resin-free mortar (with a maximum width of 90 μm, (see Figure 1A) are completely absent in the case of the hybrid mortars, which appear completely crack free and compact. All the mixtures show a very similar texture, as observed under the optical microscope (L66 and G66 not shown).

**Figure 2:**
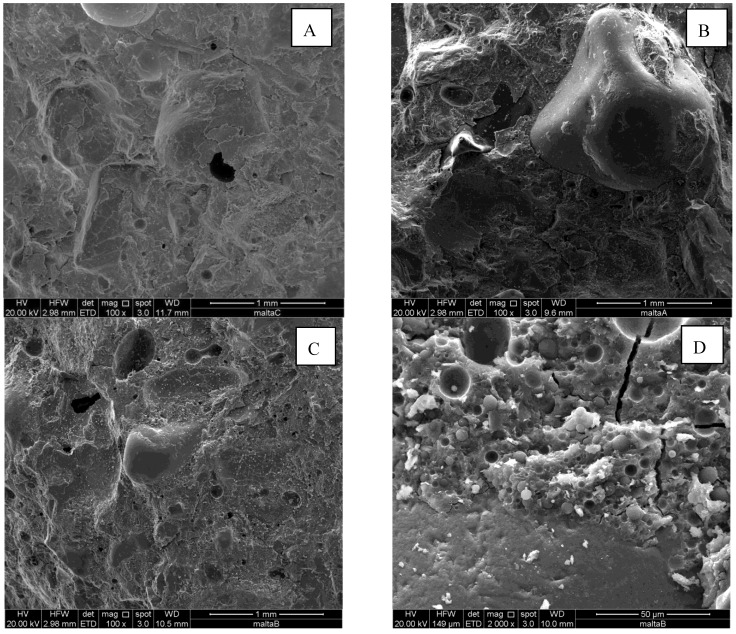
SEM micrographs of the neat geopolymer mortar (**A**) G66; (**B**) E66; and (**C**,**D**) L66.

Figure 2 shows SEM micrographs of freshly obtained fracture surfaces of the neat geopolymer mortar G66 (A), and of the specimens E66 (B) and L66 at different magnifications (C,D). In particular, Figure 2A shows the microstructure of the neat geopolymer mortar, in which the amorphous geopolymer phase incorporates the sand particles. The microstructure is dense and compact and is characterized by the presence of a glassy geopolymeric matrix. A few pores are visible, due to air bubbles incorporated during the mixture preparation. The geopolymer glassy matrix shows some minor crystals likely due to unreacted metakaolinite or residual kaolinite already present in the starting metakaolin.

The microstructure of E66 and L66 samples are very similar to each other and show that the organic phase is very homogeneously distributed in the geopolymer matrix (Figure 2D), as micrometric spherical particles of various sizes up to 50 μm. For both specimens, no agglomeration phenomena were observed. In all the cases, there is a good adhesion between the geopolymeric paste and the small size aggregate particles. For example, the strong adhesion is evidenced in Figure 2B, by the presence of some residual of the geopolymeric binder remained adhered on the sand particle when the specimens were broken to prepare the SEM samples, or in Figure 2D where no discontinuity is observable between the sand particle and the composite matrix.

The simultaneous presence of aggregates and organic resin within the specimens is expected to produce a sort of crack deflection mechanism typical of particle reinforced ceramic matrix composites, preventing the crack growth and propagation [36,46,48,49]. In this way, the mechanical properties and the fracture toughness of the geopolymer mortar are enhanced (see next sections).

#### 2.2.2. Thermal Analysis (TGA/DSC)

**Figure 3:**
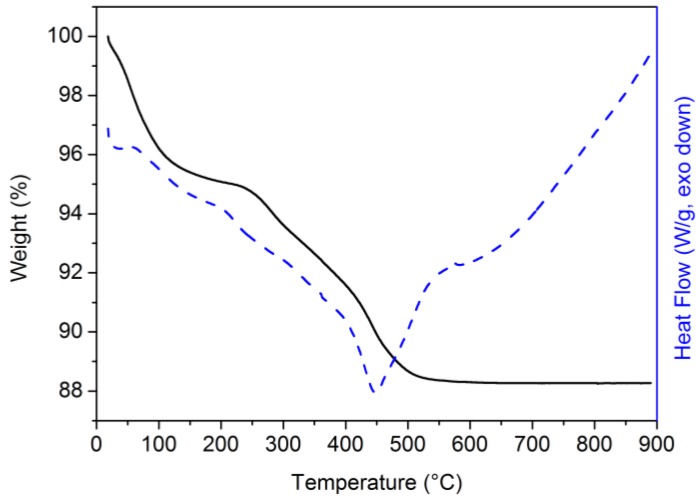
TGA (continuous line) and DSC (dashed line) curves of L66 mortar cured at room temperature for 7 days in >95% relative humidity conditions.

Simultaneous thermogravimetric and differential scanning calorimetry analyses were performed on the geopolymer mortar composites. In Figure 3 the weight loss and the thermogram for L66 are reported. Similar behavior has been found for E66 and L33/E33 (not shown) with the only difference being in the total weight loss.

The TGA and DSC curves show two principal degradation steps. The first one starts at about 30 °C and finishes at ≈180 °C and corresponds to a weight loss of ≈5%. This loss can be attributed to the removal of adsorbed water molecules or differently linked in the geopolymeric phase [50]. The second degradation process is completed at about 550 °C and corresponds to a further weight loss of ≈7%. This weight change corresponds to the degradation of the dispersed organic phase. As shown by the DSC curve, the two degradation steps are accompanied by a complex thermal behavior which main feature is an exothermic peak, centered at 450 °C.

#### 2.2.3. Water Absorption

The water absorption capacity of the three hybrid mortars G66, L66 and E66 are reported in Table 1. The addition of the resins causes a considerable decrease of WAC, even if all the mixtures show a WAC value, which is extremely low. The reduction of porosity results in lower total water absorption capacity.

**Table 1:** Water absorption capacity of tested mortars.

Mixtures	WAC (%)	Decrease
G66	2.45	1
L66	1.34	0.45
E66	1.05	0.22

#### 2.2.4. Pore Structure Characterization

**Figure 4:**
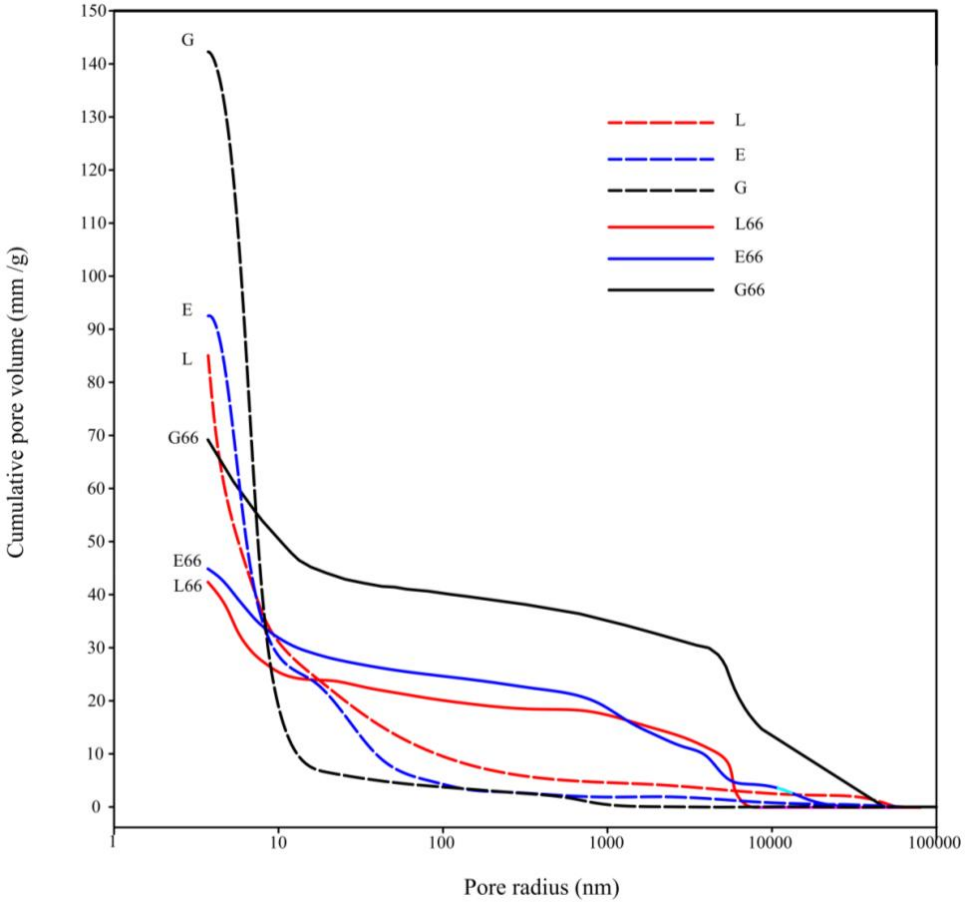
Cumulative pore volume *vs.* pore radius as obtained by mercury intrusion poroszimetry analyses for G66, L66, E66 and G, L, E specimens.

In Figure 4 the cumulative pore volume versus pore radius of the hybrid mortar specimens G66, E66 and L66 are reported, together with the analogous curves relative to the corresponding paste specimens G, E and L [46]. It is possible to observe that the curves do not have a sigmoidal shape, but show several steps. This shape indicates that the specimens are characterized by the presence of different kind of pores, according to the following classification: micropores (<0.1 μm), mesopores (0.1–1 μm) and macropores (>1 μm) [51]. Comparing G66, L66 and E66 curves, it is evident that, even if the shape of the curves is similar so indicating that the same pore size classes are present, the presence of the resin considerably lowers the total porosity. In fact, L66 and E66 specimens show a total porosity, which is about 33% lower than G66 specimen.

**Figure 5:**
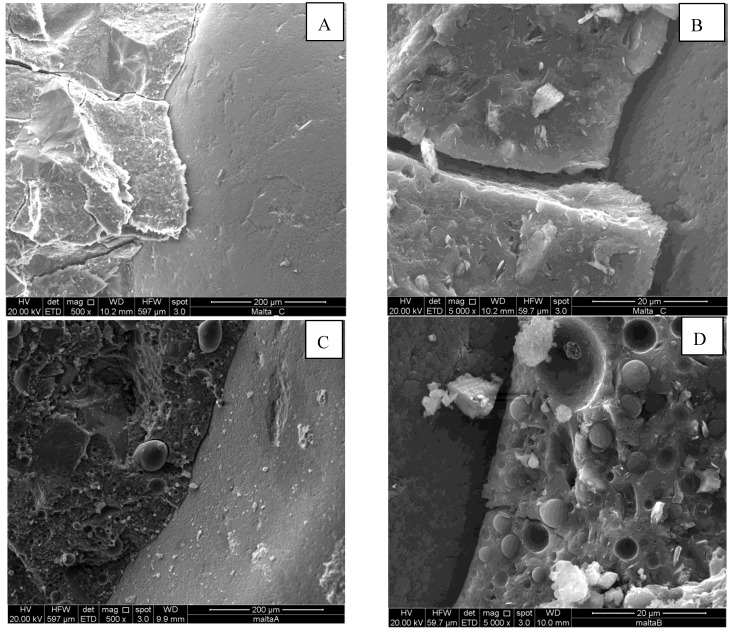
SEM micrographs of (**A**,**B**) the G66; and (**C**,**D**) E66 specimens.

By a careful examination of the figure, it can also be inferred that the addition of the aggregates has the effect of reducing up to about the 50% the total porosity of the specimens with respect to the sand-free ones. This reduction is due to the addition of the siliceous sand, which can be approximately considered as a material with very low porosity. We would have expected a still higher reduction by considering that about 70% of the mortar is constituted by sand. On the other hand, this lowering of the total porosity is accompanied by an increase of the mean pore size. This twofold effect may be interpreted with the onset of a macroporosity zone at the interface between the matrix and the sand particles of higher dimensions, similar to the Interfacial Transition Zone observed in cement and geopolymer based mortars and concretes [52,53,54]. This interpretation is corroborated by the examination of the SEM micrographs reported in Figure 5, where a clear detachment between the binding matrix and a large sand particle is observable for both the neat geopolymer G66 and hybrid composite E66 specimens. Also the size of the microcrack, about 5 μm, is consistent with literature values [52] and the porosimetric data, in fact, a sudden step is observable in the pore size distribution curve between 5 and 10 μm.

#### 2.2.5. Compressive Strength Determination

**Figure 6:**
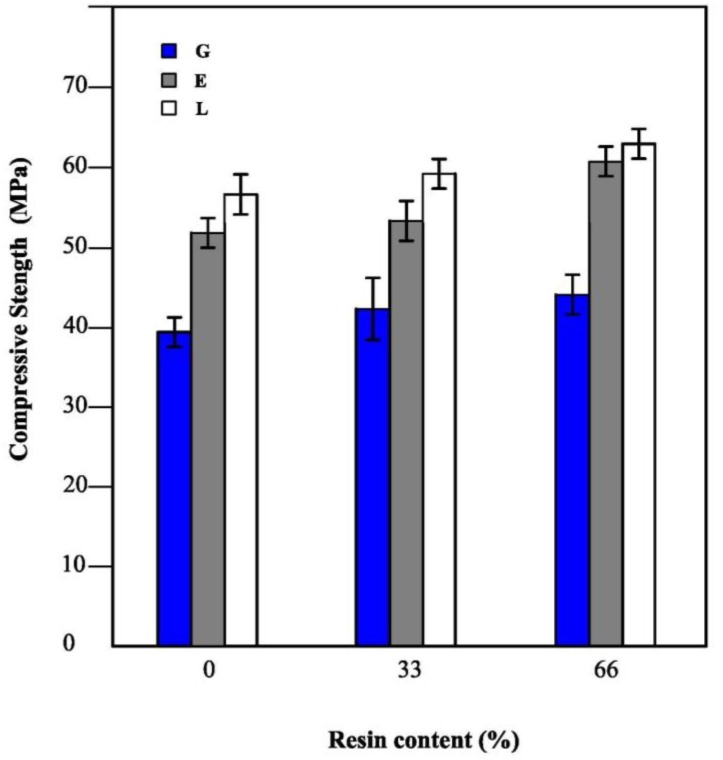
Compressive strength of G, E, L specimens as function of sand content.

The compressive strength of the mortar specimens with different sand content, prepared in the same conditions and cured for 28 days was analyzed. The obtained results are shown in Figure 6, which shows an improvement of the compressive strength of the hybrid mortars in respect to the neat geopolymeric ones. The incorporation of the organic resin in the mortar samples significantly affects the mechanical properties, as the compressive strength increases with the resin content. An explanation of this behavior likely involves the reduction of porosity reported in the previous paragraph.

Furthermore, an increase of compressive strength is also observed with the increase of the sand content. The increase in strength observed with high amount of sand can be explained by considering the introduction of a material (the sand) with higher compressive strength than the geopolymer matrix and with the reduction of the microcracking related to the drying shrinkage of the binder phase. In fact, it is well known that aggregate properties strongly affect the drying shrinkage behavior of concrete. On the other hand, the increase of strength is significantly lower than what would be expected, in fact in previous papers [42,55] an increase of about 100% was observed by the addition of a fine siliceous filler. This behavior may be likely explained by considering the onset of the Interfacial Transition Zone between the largest sand particles and the geopolymeric matrix.

The compressive strength results are in agreement with data concerning the total open porosity of the mortars. In fact, samples with high compressive strength value show lower porosity.

#### 2.2.6. Stress-Strain Response

**Figure 7:**
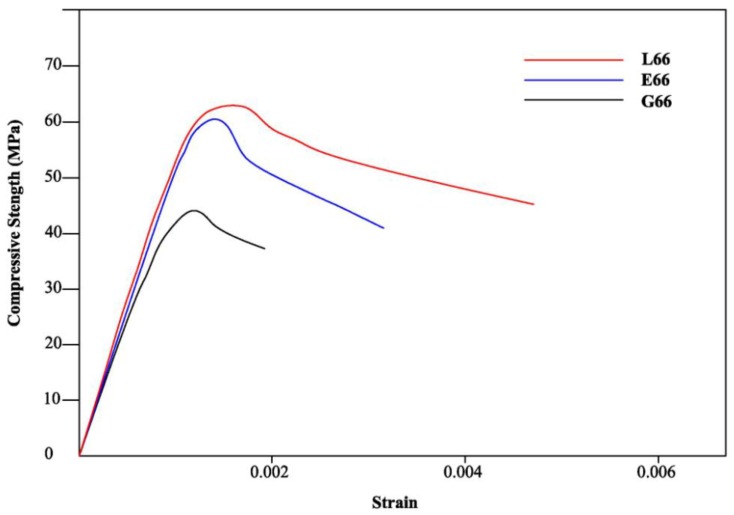
Stress-strain curves of G, G66, E, E66, L, L66 specimens.

The compressive behavior of the materials studied in this work can be examined by plotting the complete stress–strain response (Figure 7). The stress-strain curves of the hybrid mortars were plotted together with that of the geopolymer mortar. All the specimens were tested under uniaxial compression, by applying a vertical load gradually until they reached the complete failure.

From Figure 7 it is possible to establish the differences between the stress-strain relationship of a neat geopolymer mortar and a hybrid one. As already shown in the previous figure, hybrid mortar composites have higher strength than the resin free mortars. Close to the peak load, some cracks started appearing on the surface of the specimen. An examination of the stress-strain diagrams indicates that the hybrid mortars behave differently respect to the neat geopolymer one. In fact, the stress-strain curve related to composite specimens continues at high stress values after the maximum and fracture happens at high strain values. On the other side, the neat geopolymer sample fails at a strain value closer to the maximum.

The main parameters that influence stress-strain relationships are: peak stress (compressive strength value), modulus of elasticity and ultimate strain (strain at failure).

The measured peak stress (compressive strength value), and the corresponding value of strain, the ultimate strain (maximum strain of the samples) and the corresponding value of stress obtained are reported in Table 2.

The presence of epoxy resin in the mixture significantly influences the extent of all the measured parameters. Changing the kind of resin causes a slight variation in the ultimate strength, in fact EpojetLV shows a better behavior than Epojet. Moreover, the epoxy resin significantly influences the ultimate strain before cracking.

Up to now, for geopolymers, there are no structural codes. For conventional concrete, according to several codes, the ultimate strain is 3.5‰ [55,56]; for this value the compressive stress value is 60% of the compressive strength.

**Table 2:** Ultimate strain, compressive strength at ultimate strain, strain at peak stress and unconfined compressive strength of the neat geopolymer and of the hybrid composite specimens.

Mixtures	ε_ult_^a^	σ_ult_^b^ (MPa)	ε_sps_^c^	σ_sps_^d^ (MPa)
**L66**	0.0044	44.16	0.0016	61.89
**E66**	0.0028	40.09	0.0013	58.03
**G66**	0.0018	35.84	0.0012	43.16

^a^ Ultimate strain; ^b^ Compressive strength at ultimate strain; ^c^ Strain at peak stress; ^d^ Unconfined compressive strength.

Table 2 shows that L66 undergoes strains which are higher than the strains considered by code for conventional concrete. The presence of the resin in the geopolymer mortar allows the increase of strains in the descending branch and to reach high value of strain.

Even if the modulus of elasticity is similar for both hybrids, as evidenced by the linear part of the stress strain curve, the use of GeoepojetLV allows reaching higher value of peak stress and ultimate strain.

As seen, the hybrid composite specimens show progressive fracture behavior rather than a brittle one and consequently require higher fracture energy than the neat geopolymer mortar. This behavior may be likely explained by considering the propagations of cracks proceeding in a progressive and controlled way with increasing strain [57] owing to the presence of the resin phase. The organic phase may play a dual role: on one side, it could absorb part of the load by plastic deformation; on the other side, it could perform a toughening effect by a typical crack deviation mechanism [58].

## 3. Materials and Methodology

### 3.1. Materials and Design

#### 3.1.1. Materials

The epoxy resins used in this paper, Epojet^®^ and EpojetLV^®^, were purchased from Mapei S.p.A (Milan, Italy). Sodium hydroxide was purchased by Aldrich. Metakaolin, provided by Neuchem S.r.l. (San Vittore Olona, Italy), has the following composition: Al_2_O_3_ 41.90 wt %; SiO_2_ 52.90 wt %; K_2_O 0.77 wt %; Fe_2_O_3_ 1.60 wt %; TiO_2_ 1.80 wt %; MgO 0.19 wt %; CaO 0.17 wt %. The sodium silicate solution was supplied by Prochin Italia S.r.l. (Marcianise, Italy) with the composition: SiO_2_ 27.40 wt %, Na_2_O 8.15 wt % and H_2_O 64.45 wt %. Siliceous sand was employed as aggregate and its physical characteristics are shown in Table 3.

**Table 3:** Physical characteristics of siliceous sand.

Mixtures	Values	Relevant regulation
**Sand equivalent (%)**	97	EN 933-8
**Apparent density (g/cm^3^)**	2.59	MIP
**Bulk density (g/cm^3^)**	1.64	ISO 6782
**Water absorption (%)**	1.10	ISO 7033
**Fineness modulus**	2.70	UNI EN 12620

#### 3.1.2. Preparation of the Specimens

##### Preparation of the Geopolymeric Mortars (G33/G66)

The alkaline activating solution was prepared by dissolving solid sodium hydroxide into the sodium silicate solution. The solution was then allowed to equilibrate and cool for 24 h. The composition of the solution can be expressed as Na_2_O·1.4SiO_2_·10.5H_2_O. Then this was added to a mixture of MK and sand, according the amount reported in Table 4 for G33, G66, respectively.

The samples were mixed for 10 min using a planetary Hobart mixer, casted into 40 × 40 × 160 mm^3^ steel molds and vibrated for 10 min to remove air bubbles.

##### Preparation of The Hybrid Geopolymeric Mortars (E33/E66/L33/L66)

Epojet^®^ and EpojetLV^®^ resins were obtained by mixing two commercial components, named A and B, in 4:1 ratio in weight as specified in the technical data sheet supplied by the manufacturer [45].

Before being added to the geopolymeric mortar, Epojet^®^ and EpojetLV^®^ were cured at room temperature for 10 and for 60 min, respectively. Both the resins were added when they were still easily workable and long before their complete crosslinking and hardening (that takes place in about 5–7 h at 23 °C). The partially crosslinked resins were added to the freshly-prepared geopolymer mortar and quickly incorporated by mechanical mixing (10 min).

Different samples were prepared according the compositions reported in Table 4.

All the specimens in the molds were cured in >95% relative humidity conditions at room temperature for 7 days (the samples used for the mechanical tests were left further 21 days in air). The evaporation of water was prevented by sealing the top of the molds with a thin plastic layer during storage as well as during the curing stage.

Three prismatic specimens (40 × 40 × 160 mm^3^) were prepared for each mixture even if physical mechanical characterization was studied in depth only for the mixtures with high sand content, which showed better performances. For these mixtures, three additional prismatic specimens (40 × 40 × 160 mm^3^) were prepared.

**Table 4:** Mix design proportion (% wt/wt).

System	Sodium hydroxide	Sodium silicate solution	MK	Epojet	EpojetLV	Sand
G *	8.3	50.0	41.7	–	–	–
G33	5.6	33.1	28.0	–	–	33.3
G66	2.8	16.6	14.0	–	–	66.6
E *	6.7	40.0	33.3	20.0	–	–
E33	4.4	26.8	22.1	13.4	–	33.3
E66	2.2	13.4	11.1	6.7	–	66.6
L *	6.7	40.0	33.3	–	20.0	–
L33	4.4	26.8	22.1	–	13.4	33.3
L66	2.2	13.4	11.1	–	6.7	66.6

* see References [12,22].

### 3.2. Analytical Techniques

Thermogravimetric (TGA) and differential scanning calorimetry (DSC) analyses were performed by a TA Instrument SDT2960 simultaneous DSC-TGA. The analyses were performed at a heating rate of 10 °C/min, using ≈10 mg of the powdered sample under air flow.

SEM analysis was carried out by means of a FEI Quanta 200 FEG microscope.

A Controls^®^ srl (Cinisello Balsamo, Italy) crack measurement microscope (mod. 58-C0218, Magnification: 40×, Measuring range: 4 mm, Subdivision: 0.02 mm) was used for measuring crack widths. The apparatus operates by an adjustable lamp unit and the image is focused by turning a knob. The eyepiece scale can be turned through 360° to align with the direction of the crack or pitch under examination.

The pore size distribution of the prepared mixtures was measured by Mercury Intrusion Porosimetry using Pascal 140 and 440 produced by Thermo Fischer Scientific^®^ (Illkirch Cedex, France). The use of two instruments allows to determine the pore radius in the range 3.75–50.000 nm. The tests were carried out following the ISO 15901-1:2005. The total pore volume of the samples was calculated by using the well-known Washburn Equation:

(1) $$d\text{ = }\frac{-4\gamma_{\text{M}}\text{cos }\theta }{P}$$

in this formula *d* is the pore diameter (m); *γ*_M_ is the mercury surface tension (N/m); *P* is the applied pressure (MPa); and *θ* is the contact angle between the mercury and the solid surface (°).

The compressive and flexural strengths were evaluated according to EN 196-1. Mechanical test was performed in a Controls MCC8 multipurpose testing machine with a capacity of 100 kN and it was determined by taking the average of three test results. Compressive strength measurements were carried out using a Controls MCC8 hydraulic console with a capacity of 2000 kN. Each data point represents the average of six compressive strength values.

Stress-strain relationships for the casted specimens were obtained by testing mortar specimens according to EN 196-1 regulation. The tests were performed after 28 days of curing at room temperature.

Water absorption is defined as the ratio between the weight of water absorbed by a material and the weight of the dry material. Water absorption of the mortar depends on various factors like mix proportion, open porosity, type of binders, etc. Standard codes of practice give procedures to determine water absorption of the mortar. In this study UNI EN 13755:2008 was followed. The water absorption capacity (WAC) was determined as described in [59]. The water saturated specimens were dried at 40 ± 2 °C until constant weight was reached. Then, WAC was determined as follows:

(2) $$WAC\text{ = }\frac{\text{100 }(m_{w}-m_{d})}{m_{d}}$$

where *m_w_* is the water saturated specimens mass and *m_d_* is the 40 ± 2 °C dried specimens mass.

## 4. Conclusions

The present paper reports for the first time the preparation and characterization of geopolymeric mortars containing epoxy resins. The composites are produced by the addition of polymerizable commercially available epoxy monomers in liquid form to the geopolymeric mortar suspension during mixing. Epoxy-modified systems harden by the simultaneous progress of geopolymerization and epoxy polymerization. The hardened epoxy resins form spherical particles (whose diameter ranges from 1 to 50 μm) homogenously dispersed into the inorganic matrix. As a result, a co-matrix phase is formed that binds aggregates strongly.

In respect to the neat geopolymeric mortars, the geopolymeric hybrid mortars prepared present:

1. Improved strength: the polymer-modified mortars have improved compressive strength in comparison with unmodified ones. Furthermore, the polymer in the mortar helps restrain micro-crack propagation, which improves the overall toughness of the mortar.
2. The total porosity decreases with the addition of the organic polymer. This may contribute to improve gas and water impermeability and consequently the durability.

These improved properties allow the use of polymer-modified geopolymeric mortars in several applications that would otherwise be difficult or impossible, including concrete reinforcement and repair, decorative cement overlays, and many others. For these reasons polymer-modified geopolymeric mortars are promising construction materials for the future because of the good balance between their performance and cost compared to other mortar-polymer composites.
